# Maternal Anxiety Symptoms and Self-Regulation Capacity Are Associated With the Unpredictability of Maternal Sensory Signals in Caregiving Behavior

**DOI:** 10.3389/fpsyg.2020.564158

**Published:** 2020-12-22

**Authors:** Eeva Holmberg, Taija Teppola, Marjukka Pajulo, Elysia Poggi Davis, Saara Nolvi, Eeva-Leena Kataja, Eija Sinervä, Linnea Karlsson, Hasse Karlsson, Riikka Korja

**Affiliations:** ^1^FinnBrain Birth Cohort Study, Turku Brain and Mind Center, Institute of Clinical Medicine, University of Turku, Turku, Finland; ^2^Department of Psychology, University of Turku, Turku, Finland; ^3^Department of Child Psychiatry, Turku University Hospital and University of Turku, Turku, Finland; ^4^Department of Psychology, University of Denver, Denver, CO, United States; ^5^Department of Psychiatry and Human Behavior, University of California, Irvine, Irvine, CA, United States; ^6^Centre for Population Health Research, University of Turku and Turku University Hospital, Turku, Turku, Finland; ^7^Department of Psychology and Speech-Language Pathology, Turku Institute for Advanced Studies, University of Turku, Turku, Finland; ^8^Charité Universitätsmedizin Berlin, a Corporate Member of Freie Universität Berlin, Humboldt – Univeristät zu Berlin, Berlin, Germany; ^9^Department of Psychiatry, University of Turku and Hospital District of Southwest Finland, Turku, Finland

**Keywords:** maternal care, unpredictability, anxiety symptoms, depressive symptoms, self-regulation

## Abstract

The unpredictability of maternal sensory signals in caregiving behavior has been recently found to be linked with infant neurodevelopment. The research area is new, and very little is yet known, how maternal anxiety and depressive symptoms and specific parental characteristics relate to the unpredictable maternal care. The aims of the current study were to explore how pre- and postnatal maternal anxiety and depressive symptoms and self-regulation capacity associate with the unpredictability of maternal sensory signals. The study population consisted of 177 mother-infant dyads. The unpredictability of the maternal sensory signals was explored from the video-recorded mother-infant free play situation when the infant was 8 months of age. Pre- and postnatal anxiety and depressive symptoms were measured by questionnaires prenatally at gwks 14, 24, 34, and 3 and 6 months postpartum. Maternal self-regulation capacity, a trait considered to be stable in adulthood, was assessed using adult temperament questionnaire when the infant was 12 months of age. We found that elevated prenatal maternal anxiety symptoms associated with higher unpredictability in the maternal care while depressive symptoms were unrelated to the unpredictability of maternal care. Moreover, the association was moderated by maternal self-regulation capacity, as higher anxiety symptoms during pre-and postnatal period were associated more unpredictability among the mothers with low self-regulation capacity. The combination of higher amount of maternal anxiety symptoms and lower self-regulation capacity seems to constitute specific risk for the unpredictable maternal care.

## Introduction

An extensive previous literature on parenting proposes that the quality of maternal care such as maternal sensitivity to child’s cues and child secure attachment are crucial determinants for child development and are also related to various health outcomes (Bowlby, 1969; Ainsworth et al., 1978; Sroufe, 2005; Easterbrooks et al., 2012; Groh et al., 2012). Continuous and emotionally predictable care have long been considered to be key elements facilitating secure attachment pattern in the child (Bowlby, 1969; Ainsworth et al., 1978; Lyons-Ruth et al., 2019). Previous studies evaluating moment-to-moment interactions between mother and the infant support also the importance of microlevel dyadic synchrony and coordination in mother-infant early interaction (Feldman, 2007; Reck et al., 2011; Riva Crugnola et al., 2016).

A novel paradigm in the parenting research suggests that in addition to the emotional quality and synchrony of the care, the patterns of the caregiving signals *per se*, are also crucial for child brain development (Davis et al., 2019, 2017; Vegetabile et al., 2019). By patterns are hereby referred to the consistency in the order of the maternal sensory signals. Maturation of visual, auditory and somatosensory brain circuits are known to require a modality-specific signals e.g., light during sensitive period for the visual system to develop (Khazipov et al., 2004; Espinosa and Stryker, 2012; Takesian et al., 2018) and it has been assumed that specific patterns of sensory signals would be related to the development of emotional and cognitive systems as well. In current approach, the unpredictability of maternal sensory signals in caregiving behavior is analyzed at a microlevel based on the predictability of the transitions between maternal auditory, visual and tactile signals (Davis et al., 2017; Vegetabile et al., 2019). Animal studies have demonstrated that inducing stress with an impoverished bedding conditions, dams exhibit unpredictable behavior during interactions with their pups. This, in turn, has been found to associate with decreased memory functions and anhedonia in pups (Brunson et al., 2005; Ivy et al., 2008; Rice et al., 2008; Molet et al., 2016). Recently, in humans, unpredictable maternal caregiving signals were found to be associated with decreased child cognitive functioning (Davis et al., 2017), child poorer effortful control (Davis et al., 2019) and infant’s blunted cortisol response (Noroña-zhou et al., 2020). To date, little research exists, how maternal anxiety and depressive symptoms and maternal characteristics relate to the unpredictable care.

Maternal postpartum anxiety and depression are known to be connected with negative features in postnatal caregiving behavior, such as lower sensitivity, weaker structuring (Feldman et al., 2009; Field, 2010; Hakanen et al., 2019) and poorer dyadic emotional regulation (Reck et al., 2011; Riva Crugnola et al., 2016). Recently, prenatal maternal anxiety and depression have also received more and more attention in parenting studies. Prenatal anxiety is reportedly related to less optimal postnatal parenting such as more intrusive caregiving behavior (Parfitt et al., 2013; Hakanen et al., 2019). Similarly, prenatal depression has found to been linked to decreased maternal sensitivity and responsiveness in postnatal interaction (Kemppinen et al., 2006; Field, 2017). However, there is only sparse knowledge how maternal anxiety and depressive symptoms are linked with the unpredictability of maternal signals in caregiving.

Recently, maternal self-regulatory capacity has gained interest as a relevant characteristic of caregiving behavior (Crandall et al., 2015; Bridgett et al., 2017). Individual self-regulation capacity has a biological basis and refers to the top-down regulation of emotion, cognition and behavior relatively stable from the middle childhood onward (Rothbart and Bates, 2006; Laverdière et al., 2010; Putnam, 2011; Tortella-Feliu et al., 2013). One aspect of “top-down” self-regulation is effortful control that includes the ability to voluntarily inhibit, activate or change attention and behavior according to situational needs (Rothbart and Bates, 2006; Eisenberg et al., 2011). Optimal effortful control is thought to be flexible and willfully modulated and is associated with efficient coping strategies and stress regulation (Eisenberg et al., 2011). Expectedly, higher maternal effortful control is linked with greater maternal time spent in interactive caregiving activities with their 6 month-old infants (Bridgett et al., 2011) and to less negative maternal parenting behavior with 18 month-old infants (Bridgett et al., 2013).

The aspects of self-regulation also have well-established associations with psychosocial distress, including depression (Nishiguchi et al., 2016) and anxiety (Goodwin et al., 2017; Park and Moghaddam, 2017). On the other hand, high self-regulation capacity has been shown to protect from depressive and anxiety symptoms (Stevens et al., 2015; Marchetti et al., 2018), but it may also be that states like depression, fatigue and negative emotionality and self-regulation interact to predict behaviors (Baumeister et al., 2006; Feltman et al., 2009; Pomp et al., 2013). Maternal self-regulation capacity has thus been suggested to be one important moderator of the association between maternal mental health and the quality of caregiving behavior (Stein et al., 2012; Stevens et al., 2015), predisposing especially the parents with poorer self-regulation and high stress to suboptimal parenting behavior. However, to the best of our knowledge, there are no earlier studies, to date, that would investigate whether maternal self-regulation is related to the unpredictability of maternal sensory signals in caregiving. Moreover, there is little research focusing on the moderating role of self-regulation on the association between maternal anxiety and depressive symptoms and parenting, more so of the novel measure of unpredictability of caregiving.

The aim of the current study was to explore, in mothers with their 8 month old children, (1) whether the amount of maternal pre- and postnatal anxiety and depressive symptoms and maternal self-regulation capacity are related to the unpredictability of maternal caregiving behavior (2) and whether maternal self-regulation moderates this potential association. Based on previous literature (Feldman et al., 2009; Field, 2010, 2017; Bridgett et al., 2015; Stevens et al., 2015; Hakanen et al., 2019) we expected (1) that higher pre- and postnatal anxiety and depressive symptoms would be related to higher unpredictability in caregiving behavior. Specifically, we anticipated that the higher unpredictability would be most prevalent in mothers experiencing elevated levels of anxiety or depressive symptoms in any pre- or postnatal time point during the follow-up (2) that higher self-regulation capacity would be associated with lower unpredictability in caregiving behavior and (3) that self-regulation capacity would moderate the association between anxiety and depressive symptoms and unpredictability in caregiving behavior, mothers with poorer self-regulation and higher anxiety or depressive symptoms showing higher unpredictability of caregiving.

## Materials and Methods

### Study Design and Participants

The group of mother-infant pairs included in this study were a subsample of families participating in the FinnBrain Birth Cohort Study in South-Western Finland (Karlsson et al., 2018). Recruitment took place at the first trimester ultrasound between December 2011 and April 2015 at gestational week (gwk) 12. Main purpose of the study project is to investigate the effects of early life stress on child development. The Ethics Committee of the Hospital District of Southwest Finland approved the study protocol.

From the main cohort a distinct nested case-control population i.e., the Focus Cohort was drawn comprising of mothers who self-reported high levels of prenatal anxiety or depressive symptoms (cases) vs. lower levels of prenatal anxiety and depressive symptoms (controls) and their infants. The Focus Cohort criteria (Karlsson et al., 2018) for cases of prenatal distress and the cut-off points for the approximately highest and lowest 25th percentiles of maternal prenatal distress during pregnancy were established. The total sum score for the cut-off points for “cases” and “controls” were as follows: ≥ 12 and ≤ 6 for the Edinburgh Postnatal Depression Scale (EPDS) (Cox et al., 1987), ≥ 10 and ≤ 4 for the Symptom Checklist-90 (SCL-90) (Derogatis et al., 1973) anxiety subscale, and ≥ 34 and ≤ 25 points for Pregnancy Related Anxiety Questionnaire Revised 2 (PRAQ-R2) (Huizink et al., 2015). The criteria for being classified as a case were: scoring at least once above the selected threshold on two different questionnaires; or scoring at least twice above the selected threshold on the same instrument during pregnancy.

The mother-child interaction study was conducted as a part of the FinnBrain Child Development and Parental Functioning Lab assessments when the child was 8 months old (age corrected for prematurity). Study logistics and other factors unrelated to the study subjects, 354 families from the Focus Cohort were contacted during the recruitment process for the visit and 58% of them participated (*n* = 204, including 77 case families and 127 controls). From the participating mothers 195, took part in the mother-child interaction evaluation. From the video-recorded play situations 180 demonstrated a high enough quality to be analyzed using the interaction coding system (see description below). Three mothers had missing values for the socioeconomic information. For the final sample of 177 mother-infant pairs, the missing data for maternal anxiety and depressive symptoms were imputed (study question 1). From this population, 123 mothers completed the self-regulation questionnaire (study questions 2 and 3).

### Measures

#### Measuring the Degree of Unpredictability of the Maternal Sensory Signals

##### Observation of mother-infant interaction

Mother-infant interaction was video-recorded in a 10 min free-play situation. For the video recording, the mother-infant pair was placed on a soft mat with age-appropriate toys. The instructions given to the mother prior to the video recording were: “This is a free-play time with your infant. You can use the toys, or you can play without the toys. Try to play as you are used to playing with your infant at home.”

##### Coding maternal sensory signals

From the videotapes, maternal sensory signals, including auditory, visual, or tactile sensory signals to the child were coded continuously in real time using The Observer XT 11 (Noldus). Auditory stimuli included all auditory signals that the mother provided for the child during the play session, e.g., speech and laughter. The visual signals were all the objects that the mother presented and provided for the child e.g., showing a toy. Moreover, the analysis included whether the child followed the mother’s visual signals, for example looked at the toy that the mother was showing. Tactile signals were touching, stroking or holding the child. Descriptions for the behavioral codes used to characterize maternal sensory input to her child are presented in Table 1. Coders were unaware of the mother’s background information and 10% of the tapes were double scored. Interrater agreement (%) between the two main coders was 86.1%. Additional information on the details of behavioral coding and training manual are found^1^.

**TABLE 1:** Description of behavioral codes used to characterize maternal sensory input to her child.

**Sensory category**	**Behaviors**	**Description**
Auditive	Maternal vocalizations	Mother makes a vocalization (“There is a ball. The ball is red.” There are two distinct vocalizations)
Tactile	Maternal touch	Touching, stroking
	Maternal holding	Holding the child
Visual	Mother manipulating objects	Mother is holding a toy or other object
	Child visually attending to maternal activities	Child is looking at a toy mother is manipulating

##### Quantifying unpredictability of maternal sensory signals (entropy rate)

After coding the maternal sensory signals, the unpredictability of the maternal sensory signals (defined as an entropy rate) was calculated using an R 3.6.1.

The codes for maternal sensory signals were not mutually exclusive (i.e., mother could also provide two or three signals simultaneously). There were thus eight possible combinations between maternal sensory signals (see Table 2). Changes between any of the 8 behaviors or combinations of behaviors were identified as transitions. For example, a mother might be speaking to the child while showing her a toy (auditory and visual input). If she additionally picked up the child (tactile input) so that she now provides auditory, visual, and tactile input, then this would be considered a transition from “auditory and visual” combination to “all signals” combination.

**TABLE 2:** Combinations between maternal sensory signals.

No behavior	No auditory, tactile or visual signals
Single behavior	Only auditory signals Only tactile signals Only visual signals
Combination of two behaviors	Both auditory and tactile signals Both tactile and visual signals Both auditory and visual signals
Combination of all behaviors	All signals: auditory, tactile, and visual

The observed transitions were then used to estimate the transition probabilities between the combinations. The distribution of the transition probabilities then reflected the uncertainty of mother’s behavior for the observer, illustrated by the following example: If “auditory and visual” combination was always followed by “only auditory” combination, this would indicate the maximum predictability. If “auditory and visual” combination was followed by “only auditory” combination 80% of times and by “all signals” combination 20% of times, that would indicate less predictability. If “auditory and visual” combination was followed by “only auditory” combination 40% of times, by “all signals” combination 30% of times and “only visual” combination 30% of times, that would indicate even less predictability. If “auditory and visual” combination was followed by any other combination with totally randomly (i.e., with mutually equal probabilities), this would indicate minimum predictability (i.e., maximum unpredictability).

The estimated transition probabilities were then summarized by one number, i.e., the entropy rate, which describes the overall unpredictability of mother’s behavior. The entropy rate ranges from 0 to 2.807, higher values indicating more unpredictable and unsystematic caregiving behavior. Additional details regarding the calculation of the entropy rate and a description of the R software package for calculating the entropy rate are provided in Davis et al., 2017 and available at^2^.

The number of the transitions of maternal sensory signals was not associated with the entropy rate (*r* = −0.048, *p* = 0.53; number of transitions: *M* = 360.5, *SD* = 111.5, range = 45–662) indicating that entropy rate is a separate construct from the quantity of the signals.

#### Anxiety and Depressive Symptoms

Maternal prenatal anxiety and depressive symptoms were assessed at gwks 14, 24, 34, and 3 and 6 months postpartum using the Symptom Checklist-90 (SCL-90) anxiety subscale and the Edinburgh Postnatal Depression Scale (EPDS). The SCL-90 is a reliable and valid measure of anxiety symptoms in both clinical and research settings (Derogatis et al., 1973; Holi et al., 1998) and consists of 10 items rated from 0 to 4. The EPDS is a widely used measure of both prenatal and postnatal depression (Cox et al., 1987) and consists of ten items rated from 0 to 3. All measures demonstrated good internal consistency throughout the study (0.84 –0.90 for SCL-90, 0.85 –0.90 for EPDS). Anxiety and depressive symptoms were used both as continuous (prenatal, postnatal and a mean of pre- and postnatal anxiety and depressive symptoms) and categorical (highest 10th percentile cut-off at each measurement point) variables.

#### Maternal Self-Regulation Capacity

Maternal self-regulation capacity, i.e., effortful control was assessed using the Adult Temperament Questionnaire (ATQ; Evans and Rothbart, 2007) when the child was 12 months old, per previous studies showing adequate to good re-test reliability of ATQ (Laverdière et al., 2010; Tortella-Feliu et al., 2013). The ATQ includes 77 questions forming four factors: effortful control, negative affect, extraversion and orienting sensitivity. The factor of effortful control includes three subscales: activation control (7 items), attentional control (5 items) and inhibitory control (7 items). Both the effortful control factor and its subscales were used in the analyses as the continuous variables. Activation control is the ability to perform a task despite a lack of desire to engage in the activity; attentional control refers to the ability to remain focused on a task; and inhibitory control involves suppressing an inappropriate response. The effortful control factor and its subscales showed adequate internal consistency in this study (0.82 for effortful control, 0.62 –0.70 for the subscales).

### Statistical Analysis

SPSS 24 and PROCESS Macro v 3.4 were used for the statistical analyses. First, attrition was examined with independent *t*-tests, U-tests and Chi-square statistics. The unpredictability of maternal caregiving behavior and effortful control were normally distributed, and thus, even though, the distributions of anxiety and depressive symptoms were skewed, use of linear regression modeling was allowed. Missing data for anxiety (SCL-90) and depressive (EPDS) symptoms (for the prenatal symptom measurements < 5% and for the postnatal measurements < 17%) were imputed using the random forest-method (MissForest) (Stekhoven and Bühlmann, 2012) in the R 3.6.1. In the imputation of each symptom variable, the symptom variables at the other time points were used as the predictors. Two respondents with no data according to the questionnaires were removed from the analysis.

The associations between background variables and the unpredictability of maternal sensory signals were analyzed with ANOVA, *t*-test and Pearson’s correlations. Background variables associated with the unpredictability of maternal sensory signals were included as covariates in the following models. Associations between anxiety and depressive symptoms and the unpredictability of maternal sensory signals were analyzed with Spearman correlations and *p*-values for 10 comparisons were corrected using Benjamini-Hochberg correction and FDR < 0.10 threshold.

To examine associations between elevated levels of anxiety and depressive symptoms and maternal unpredictability, cut-offs for maternal symptoms were formed on the basis of the highest 10% percentile in each measurement point (for SCL-90 gwk 14 ≥ 10, gwk 24 ≥ 13, gwk 34 ≥ 11, 3 months ≥ 10, 6 months ≥ 10 and for EPDS gwk 14 ≥ 12, gwk 24 ≥ 13, gwk 34 ≥ 13, 3 months ≥ 11, 6 months ≥ 12). A covariance analysis ANCOVA was conducted to test the main effects of the elevated anxiety and depressive symptoms explaining the unpredictability of maternal sensory signals. The *p*-values of each symptom predictor across all 10 models were corrected using Benjamini-Hochberg correction and FDR < 0.10 threshold due to the high correlation of predictors in each model.

Finally, associations between the self-regulation capacity and the unpredictability of maternal sensory signals were examined with Pearson correlations. *P*-values for 4 comparisons were corrected using Benjamini-Hochberg correction and FDR < 0.10 threshold. Associations between maternal self-regulation capacity and anxiety and depressive symptoms were examined with Spearman correlations. Subsequently, to study the possible interaction effect of maternal anxiety or depressive symptoms and self-regulation capacity on the unpredictability of maternal sensory signals, a linear regression model was conducted separately for anxiety and depressive symptoms and each aspects of maternal self-regulation (effortful control and its subscales activation control, attentional control, inhibition control). For the models, mean scores for pre- (qwks 14, 24, 34) and postnatal (3 and 6 months) anxiety and depressive symptoms were used due to the high collinearity of pre- and postnatal symptom measurements and to restrict the number of regression models. In each model, the first step included the significant covariates; anxiety or depressive symptoms and self-regulation were added in the second step of the model; and the interaction term between effortful control (or subscale of it) and anxiety or depressive symptoms was added to the third step. The *p*-values for the eight interaction terms were corrected using Benjamini-Hochberg method and FDR < 0.10 threshold was used due to the correlation between the dependent variables in the models. Simple slope analysis was conducted using PROCESS Macro v3.4.

## Results

### Sample Characteristics and Attrition

Demographic characteristics of the sample (*n* = 177) are presented in Table 3. The mothers who did not participate to the 8 month-visit (*n* = 159) did not significantly differ from the participants (*n* = 195) with regards to pre- or postnatal anxiety and depressive symptoms, parity or monthly income level (*p* > 0.05), but they had a lower education level [χ^2^(2) = 14.07, *p* = 0.001] and were younger [t (313) = 2.35, *p* = 0.020] compared to the participating mothers.

**TABLE 3:** Sample characteristics (*n* = 177).

	**Sample *n* = 177**	
	**n (%)**	**Mean (SD)**
**Education**		
High school (at least)	42 (23.7)	
Polytechnics	67 (37.9)	
University	68 (38.4)	
Monthly income*		
1,500 or less	60 (33.9)	
1,501–2,500	100 (56.5)	
>2,500	17 (9.6)	
Maternal age		31.18 (4.01)
Primiparous	103 (58.2)	
Unpredictability of maternal sensory signals		0.90 (17)
*theoretical range* 0–3		
**Maternal effortful control**		
*theoretical range* 1–7		4.62 (0.74)
**Distress symptoms**		
**First trimester**		
SCL-90		3.53 (4.40)
*theoretical range* 0–40		
EPDS		4.98 (4.40)
*theoretical range* 0–30		
**Second trimester**		
SCL-90		4.42 (5.40)
EPDS		5.03 (4.92)
PRAQ-R2		22.51 (7.19)
*theoretical range* 10–50		
**Third trimester**		
SCL-90		3.45 (4.72)
EPDS		5.04 (4.90)
PRAQ-R2		22.52 (7.04)
**Postpartum 3 months**		
SCL-90		3.15 (4.04)
EPDS		4.60 (4.13)
**Postpartum 6 months**		
SCL-90		3.60 (4.95)
EPDS		5.20 (5.00)
**SCL-90 pre- and postnatal**		
Mean		3.63 (4.05)
**EPDS pre- and postnatal**		
Mean		4.97 (3.95)

*in Euros.

The mothers who did not complete the ATQ questionnaire (*n* = 54) did not statistically significantly differ from mothers who responded to the questionnaire with regard to monthly income, age, parity or postnatal anxiety and depressive symptoms (*p* > 0.05). However, the non-respondent mothers had a significantly lower education level [χ^2^(2) = 6.60, *p* = 0.037], a higher number of prenatal anxiety and depressive symptoms [χ^2^(1) = 3.92, *p* = 0.048] and a higher unpredictability [*t* (175) = 2.52, *p* = 0.013] than the respondent mothers. The mean for the unpredictability of maternal sensory signals for the respondents was *M* = 0.88 (0.17) and for the non-respondents *M* = 0.94 (0.17).

### Background Variables and the Unpredictability of Maternal Sensory Signals

Maternal education level related significantly to unpredictability of maternal sensory signals [*F*_(2,174)_ = 3.520, *p* = 0.032]: mothers of the middle education group (polytechnics) were significantly more unpredictable in their care, M (SD) = 0.94 (0.17) compared to the mothers in the high education group (university), M (SD) = 0.86 (0.15), *p* = 0.011. Lower monthly income level (*r* = −0.160, *p* = 0.033) and lower maternal age (*r* = −0.184, *p* = 0.014) were also found to be associated with higher maternal unpredictability. Gestational weeks, birth weight, gender of the child and parity did not relate significantly to the unpredictability of maternal signals. Subsequently, maternal education, income and age were chosen as covariates in the final analyses.

### Main Effects of Anxiety and Depressive Symptoms on the Unpredictability of Maternal Sensory Signals

The observation of pre- and postnatal periods using continuous variables showed that only higher maternal anxiety symptoms 3 months postpartum correlated significantly with the higher unpredictability of maternal sensory signals (*r* = 0.148, *p* = 0.049, FDR = 0.49). The examination of time points with categorical classifications (highest 10th percentile) revealed that higher unpredictability was exhibited by the mothers reporting elevated levels of anxiety symptoms at gwk 24 vs. mothers with lower symptoms [*t* (175) = −2.87, *p* = 0.005, FDR = 0.05], but other differences between the percentile groups were not observed (see Table 4). No associations were found between elevated depressive symptoms and the unpredictability of maternal sensory signals. Also, pre- and postnatal anxiety and depressive symptom mean scores were not related to the unpredictability of maternal sensory signals.

**TABLE 4:** Differences in the unpredictability of maternal sensory signals between elevated (highest 10th percentile) and lower level (90th percentile) of anxiety and depressive symptom groups (*n* = 177).

	**Anxiety (SCL-90)**		**Depression (EPDS)**	
	
	**M (SD) n**	**t (df)**	**M (SD) n**	**t (df)**
gwk 14 highest 10th percentile 90th percentile	0.93 (0.16) 22 0.89 (0.17) 155	−1.04 (175)	0.90 (0.16) 19 0.90 (0.17) 158	−0.08 (175)
gwk 24 highest 10th percentile 90th percentile	1.00 (0.18) 20 0.88 (0.17) 157	−2.87 (175) **	0.92 (0.16) 18 0.89 (0.17) 159	−0.56 (175)
gwk 34 highest 10th percentile 90th percentile	0.93 (0.18) 23 0.89 (0.17) 154	−0.99 (175)	0.91 (0.18) 18 0.89 (0.17) 159	−0.42 (175)
3 months highest 10th percentile 90th percentile	0.92 (0.19) 20 0.89 (0.17) 158	−0.53 (175)	0.95 (0.19) 18 0.89 (0.17) 159	−1.48 (175)
6 months highest 10th percentile 90th percentile	0.92 (0.21) 19 0.89 (0.17) 158	−0.71 (175)	0.90 (0.18) 17 0.90 (0.17) 160	−0.16 (175)

*p < 0.05, **p < 0.01.

Covariance analysis (ANCOVA) was conducted to test whether the association between elevated prenatal anxiety symptoms (gwk 24) and the unpredictability of maternal signals remained significant after socioeconomic factors were controlled for. Higher levels of prenatal anxiety symptoms (β = 0.197, *p* = 0.007) remained related to higher unpredictability after controlling for socioeconomic factors. The level of education (β = 0.173, *p* = 0.035) and a younger maternal age (β = −0.168, *p* = 0.028) were also significantly associated with higher unpredictability (see Table 5).

**TABLE 5:** ANCOVA for the unpredictability of maternal sensory signals (*n* = 177).

	**F_(df)_**	**β**	**R^2^**
Adjusted model	4.844_(5,171)_**		0.098
Highest 10th percentile prenatal anxiety	7.408_(1,171)_*	0.197**	
Education level Low vs. high Middle vs. high	4.082_(2,171)_*	−0.061 0.173*	
Monthly income	1.573_(1,171)_	−0.096	
Maternal age	4.908_(1,171)_*	0.168*	

*p < 0.05, **p < 0.01.

### Main Effect of Maternal Self-Regulation Capacity on the Unpredictability of Maternal Sensory Signals

Effortful control was not related to the unpredictability of maternal sensory signals. From the subscales lower attentional control correlated significantly with the higher unpredictability of maternal sensory signals (*r* = −0.180, *p* = 0.046, FDR = 0.116).

### The Interaction Between Maternal Anxiety and Depressive Symptoms and Maternal Self-Regulation in Predicting the Unpredictability of Maternal Sensory Signals

The interaction effects of pre- and postnatal anxiety and depressive symptom mean scores and the effortful control and its subscales on the unpredictability of maternal sensory signals were examined next. All four scales of maternal self-regulation capacity were related to lower anxiety and depressive symptoms throughout pre- and postnatal period and pre- and postnatal mean symptom scores (Table 6).

**TABLE 6:** Spearman correlations between maternal anxiety and depressive symptoms and self-regulation capacity (*n* = 123).

	**Effortful control**	**Activation control**	**Attentional control**	**Inhibition control**
SCL-90 gwk 14	−0.387**	−0.323**	−0.307**	−0.349**
SCL-90 gwk 24	−0.407**	−0.294**	−0.357**	−0.358**
SCL-90 gwk 34	−0.448**	−0.329**	−0.386**	−0.409**
SCL-90 3 months	−0.340**	−0.293**	−0.266**	−0.269**
SCL-90 6 months	−0.454**	−0.388**	−0.390**	−0.352**
SCL-90 pre-and postnatal mean	−0.495**	−0.388**	−0.419**	−0.421**
EPDS gwk 14	−0.384**	−0.319**	−0.327**	−0.302**
EPDS gwk 24	−0.375**	−0.282**	−0.309**	−0.300**
EPDS gwk 34	−0.413**	−0.311**	−0.331**	−0.345**
EPDS 3months	−0.486**	−0.402**	−0.401**	−0.352**
EPDS 6 months	−0.514**	−0.427**	−0.429**	−0.378**
EPDS pre- and postnatal mean	−0.525**	−0.430**	−0.439**	−0.392**

*p < 0.05, **p < 0.01.

We did not observe a significant interaction between effortful control and anxiety or depressive symptoms in predicting the unpredictability of maternal sensory signals. However, when focusing on the subscales there was a significant interaction of activation control and anxiety symptoms in predicting the unpredictability of maternal signals (β = −1.074, *p* = 0.008, FDR = 0.064) (see Table 7). The model explained 9.1% of the variance. The interactions were not statistically significant for the subscales of attentional control and inhibitory control.

**TABLE 7:** Standard linear regression model for the unpredictability of maternal sensory signals (*n* = 123).

	**β**	**R^2^ (adj)**	**ΔR^2^**
Step 1		0.019	0.052
Education			
Low vs. high	−0.064		
Middle vs. high	0.167		
Monthly income	−0.084		
Maternal age	−0.037		
Step 2		0.041	0.036
Education			
Low vs. high	−0.072		
Middle vs. high	0.175		
Monthly income	−0.006		
Maternal age	−0.002		
Pre- and postnatal anxiety mean	0.079		
Activation control	−0.150		
Step 3		0.091*	0.055**
Education			
Low vs. high	−0.068		
Middle vs. high	−162		
Monthly income	−0.042		
Maternal age	−0.104		
Pre- and postnatal anxiety mean	1.182**		
Activation control	0.049		
Pre- and postnatal anxiety mean * activation control	−1.074**		

*p < 0.05, **p < 0.01.

**FIGURE 1:**
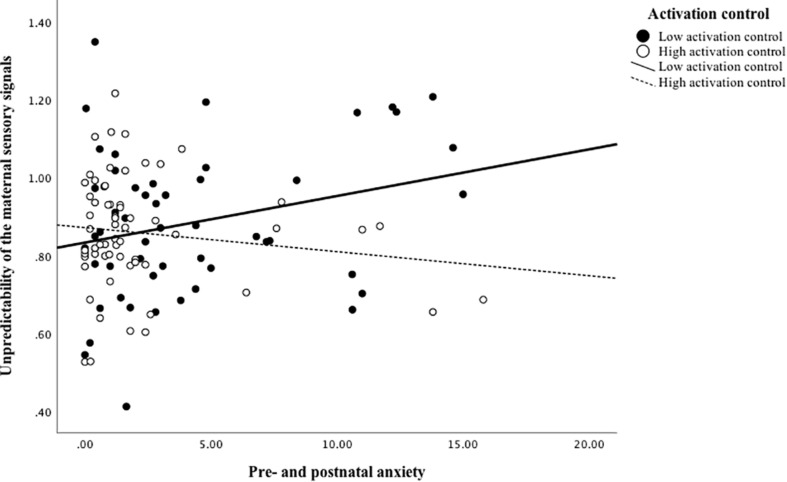
Maternal anxiety predicting for the unpredictability of maternal sensory signals in low and high activation control groups (groups divided by median value 4.57 of activation control).

Simple slope analysis for the maternal anxiety symptoms and the unpredictability of maternal sensory signals amongst those with low (−1SD), average (0SD) and high (+ 1SD) activation control revealed that the association between maternal anxiety symptoms and unpredictability was significant only when the level of maternal activation control was low (β = 0.009, 95% CI [0.001, 0.018], *p* = 0.033, p adjusted for covariates = 0.062), but not when the level of activation control was average [β = −0.001, 95% CI = (−0.009, 0.008), *p* = 0.905] or high [β = −0.011, 95% CI (−0.024, 0.003), *p* = 0.129]. See Figure 1 for illustration of the interaction effect.

## Discussion

This study explored the association of maternal anxiety symptoms, depressive symptoms, and self-regulation capacity on the unpredictability of maternal sensory signals in caregiving behavior. In addition, the study explored whether maternal self-regulation moderated the association between maternal anxiety and depressive symptoms and unpredictability of maternal sensory signals. A novel method was used to analyze the unpredictability of maternal sensory signals at a microlevel in the mother-child interaction (Davis et al., 2017; Vegetabile et al., 2019). We found that elevated maternal prenatal anxiety (gwk 24) but not postnatal anxiety symptoms associated with higher unpredictability in maternal caregiving behavior after controlling for socioeconomic factors. Maternal self-regulation capacity was not significantly associated with the unpredictability of caregiving after adjustments, but interestingly, maternal self-regulation capacity moderated the association between anxiety symptoms during pre- and postnatal period and higher unpredictability of the maternal care. Thus, mothers with poorer self-regulation and higher levels of anxiety symptoms exhibited more unpredictability of caregiving. To our knowledge, this study is among the first exploring maternal anxiety and depressive symptoms and maternal characteristics explaining the unpredictability of maternal sensory signals in caregiving behavior.

An important finding in our study was that anxiety symptoms, as a hypervigilant state (Glover, 2011; Goodwin et al., 2017), were associated with more unpredictability in the maternal caregiving. Depressive symptoms, which are more associated with the passive dimensions of behavior, were not found to associate with unpredictability. The result is in line with the rat models, in which an impoverished bedding condition was used to evoke stress and unpredictable behavior in the dam (Ivy et al., 2008; Rice et al., 2008). Anxiety is known to distract attentional capacity which in turn has been found to be related to disruptions in mother-child interactions such as increased maternal control (Stein et al., 2012). Moreover, the result is in line with previous studies suggesting that anxiety may have more negative impact for the quality of mother-child interactions than depression (Parfitt et al., 2013; Riva Crugnola et al., 2016; Hakanen et al., 2019). In our study, only elevated prenatal anxiety symptoms were significantly related to unpredictable care, even though there was a trend level relation between elevated anxiety symptoms and unpredictable care throughout pre-and postnatal period. Prenatal anxiety symptoms may have biological effects on fetal development via prenatal programming which may affect the quality of mother-child interaction (Sandman et al., 2011). Prenatal anxiety may also affect via psychological mechanisms by interfering prenatal parenting (Dayton et al., 2010; Ahlqvist-Björkroth et al., 2016). Moreover, prenatal mood disturbances might interfere with the programming of parenting behavior (Brunton and Russell, 2008; Hillerer et al., 2014; Glynn et al., 2018) leading to difficulties in adaptation to motherhood. One possible reason for the non-significant association between elevated postnatal anxiety symptoms (highest 10th percentile) and unpredictable care may be related to the small group sizes of those with elevated anxiety symptoms at the pre- and postnatal measurement points; this may have limited the power for group comparisons.

Interestingly, the interaction between maternal self-regulation capacity, more specifically activation control, and anxiety symptoms throughout the pre- and postnatal periods was significantly related to the unpredictability of the maternal caregiving behavior. Higher anxiety symptoms throughout pregnancy and 3 and 6 months postpartum were associated with higher unpredictability of caregiving behavior in mothers who had lower maternal self-regulation capacity. These findings add to the growing evidence that maternal self-regulation capacity is an important characteristic to consider when studying the quality of parenting practices (Bridgett et al., 2011, 2013), and confirms our hypothesis that self-regulation actually moderates the effect of maternal distress, e.g., anxiety on caregiving quality, providing a new insight into the determinants of maternal caregiving quality.

There are several possible mechanisms explaining this finding. First, a capacity for high self-regulation may buffer the detrimental effects that maternal anxiety symptoms may have on the unpredictability of maternal caregiving behavior (Bakker et al., 2011; Stevens et al., 2015). Second, anxiety symptoms may hamper maternal self-regulation which in turn increases the unpredictability of maternal care (Liston et al., 2009; Diamond, 2013). In addition, self-regulation capacity shows moderate levels of genetic influence (Bridgett et al., 2015) which may manifest not only as lower activation control but also as higher anxiety or depressive symptoms and unpredictable caregiving behavior and this disposition appears when individuals are challenged in stressful situations. With no previous research on the potential of activation control to predict parenting, the mechanisms why the interaction with only this subscale was found remain unclear at this state. However, given that it describes the ability to perform a task despite a lack of desire to engage in the activity (Eisenberg et al., 2011), and parenting poses many such challenges for the parent, it could be hypothesized that activation control is specifically related to fluent and coherent behaviors in caregiving situations especially in the context of distress.

Although not the main foci of this study, socioeconomic factors were significantly associated with unpredictability of maternal caregiving behavior. A lower education level, monthly income and maternal age were related to higher unpredictability of caregiving behavior. The results support earlier findings addressing that low socioeconomical status and low maternal age may associate with less than optimal parenting behaviors (Conger et al., 2011; Camberis et al., 2016; Roubinov and Boyce, 2017).

Clinically, our findings strengthen the view that interventions aimed at targeting the characteristics of maternal caregiving behavior could already be initiated during pregnancy and focus comprehensively on the possibly accumulating risk factors. Our study also suggests that anxiety symptoms may be a specific risk factor for the high unpredictability of maternal caregiving behavior and should be also considered and screened for in clinical settings during pregnancy. In addition, recognition of maternal self-regulation skills before parenthood might be one possible focus of the counseling or even interventions; recently, self-regulatory processes have been shown to be linked with better maternal mentalization capacity and reflective functioning (Rutherford et al., 2018), providing one potential avenue for such treatment.

There are some limitations to be considered in this study. In general, the findings of the present study should be considered preliminary and similar to that of cross-sectional research. Maternal self-regulation was evaluated only at one time point when the child was 1 year of age, after the outcome variable. However, self-regulation capacity has shown to be stable across adulthood, providing a strong rationale to use it even if assessed after the parenting outcome (Laverdière et al., 2010; Putnam, 2011; Tortella-Feliu et al., 2013). Beyond effortful control, similar consistency is observed in the closely related self-regulation concepts, such as conscientiousness and executive functioning (Eisenberg et al., 2012; Friedman et al., 2016; Kim and Kochanska, 2020). This argument on consistency is further supported by the pattern of correlations between maternal self-regulation and maternal anxiety and depressive symptoms that remain very similar in our study across the follow up from gestational week 14 to 6 months postpartum (r’s ranging from 0.30 to 0.40) (see Table 6), even though mothers in the study undergo pregnancy and postpartum that are major periods of change in their lives. Another limitation was that the significance of the timing of the elevated anxiety and depressive symptoms could not be studied comprehensively in this study because of the small group sizes with elevated anxiety and depressive symptoms at the different measurement points. In addition, on average, the level of anxiety and depressive symptoms were somewhat modest and psychiatric diagnostic criteria were not used, which may limit the generalizability to clinical populations. Moreover, in our data sample the participating mothers had higher education and higher age compared to non-participating mothers which might have affected for the results.

It is important to notify that maternal anxiety symptoms, self-regulation and significant covariates together explained 9.1% of the variance in unpredictable maternal care. Relatively low effect size is, however, in line with previous studies exploring the associations between maternal anxiety and depressive symptoms, maternal characteristics and maternal caregiving behavior (Kim et al., 2012; Stack et al., 2012; Stevens et al., 2015; Hakanen et al., 2019). This is suggesting that maternal unpredictable care is a complex and multifactorial system affected by several factors e.g., genetics, infant and paternal characteristics (Ierardi et al., 2019). However, in our or previous studies, infant characteristics (gestational weeks, birth weight, gender of the child), were not related to unpredictable maternal signals (Davis et al., 2017, 2019). In the future, maternal characteristics in relation to the unpredictable maternal care needs to be studied more comprehensively to attain better understanding of the underlying mechanisms, to aim at more focused parenting interventions.

To our knowledge, this study was the first to report association between maternal anxiety symptoms and self-regulation capacity and the unpredictability of maternal caregiving behavior. Previous parenting studies have focused on the emotional component of parenting whereas this novel approach of the unpredictability of maternal sensory signals may capture a new aspect of the mother-child interaction.

## Data Availability Statement

The datasets presented in this article are not readily available. There are strict legal rules in terms of data sharing in the medical faculty at the University of Turku. The dataset is available upon request. Requests to access the datasets should be directed to Juho Pelto, jepelt@utu.fi.

## Ethics Statement

The studies involving human participants were reviewed and approved by the Ethics Committee of the Hospital District of Southwest Finland. Written informed consent to participate in this study was provided by the participants’ legal guardian/next of kin.

## Author Contributions

EH: writing–original draft. TT: writing–review and editing. MP: supervision, writing–review, and editing. ED and RK: supervision, writing–review, and editing, methodology. SN: investigation, writing–review, and editing, methodology. EK: investigation, writing–review, and editing. ES: investigation. HK and LK: resources, funding acquisition, conceptualization, writing–review, and editing. All authors contributed to the article and approved the submitted version.

## Conflict of Interest

The authors declare that the research was conducted in the absence of any commercial or financial relationships that could be construed as a potential conflict of interest.

## Footnotes

1 https://contecenter.uci.edu/shared-resources/

2 https://contecenter.uci.edu/shared-resources/
